# Association of Distance to Gynecologic Oncologist and Survival in a Rural Midwestern State

**DOI:** 10.1089/whr.2022.0016

**Published:** 2022-08-04

**Authors:** Keely K. Ulmer, Breanna Greteman, Megan McDonald, Jesus Gonzalez Bosquet, Mary E. Charlton, Sarah Nash

**Affiliations:** ^1^ Division of Gynecologic Oncology, Department of Obstetrics and Gynecology, University of Iowa Hospitals and Clinics, Iowa City, Iowa, USA.; ^2^ Department of Epidemiology, University of Iowa College of Public Health, Iowa City, Iowa, USA.

**Keywords:** rural, ovarian, cancer, distance, gynecologic oncology

## Abstract

**Objectives:**

Rural ovarian cancer patients experience worse survival compared to urban patients. We assessed whether distance to gynecologic oncology specialists was associated with survival for patients in a rural state.

**Methods:**

Demographic, tumor, and treatment characteristics were extracted from the Iowa Cancer Registry for patients diagnosed between 1990 and 2018. Data were linked to the county-level 2018–2019 Area Health Resource File (number of surgeons and hospital beds per 100,000 population). Rurality was defined using 2013 Rural-Urban Continuum Codes; distance to the nearest gynecologic oncologist was calculated from the centroid of the county of residence to the centroid of the nearest county with a high volume health care center with a gynecologic oncologist. Associations with survival were assessed using multivariable Cox proportional hazards models.

**Results:**

Analyses included 1,562 ovarian cancer patients. Mean distance to gynecologic oncology was 60.8 miles, and median survival was 23 months. Unadjusted models showed increased distance from gynecologic oncology had progressively greater risk of death 30–49 miles (hazard ratio [HR] = 1.09, confidence interval [CI]: 1.04–1.15), 50–69 miles (HR = 1.19, CI: 1.07–1.32), 70+ miles (HR = 1.30, CI: 1.11–1.51). In adjusted models, association of distance to gynecologic oncology with risk of death was not significant; however, more advanced cancer stage and age, unmarried status, and higher county-level poverty were independently associated with increased risk of death.

**Conclusions:**

Above and beyond demographics and stage, distance to gynecologic oncology care was not an independent predictor of ovarian cancer survival. Further studies are needed to determine how to mitigate the factors contributing to worsened ovarian cancer survival among rural patients.

## Introduction

Ovarian cancer is a rare but deadly cancer of the female reproductive tract. Each year ∼21,000 women are diagnosed with this malignancy in the United States, resulting in 15,000 deaths.^1^ Approximately 1.2% of women will be diagnosed with ovarian cancer at some point during their lifetime, and the 5-year relative survival is only 49%,^2^ making ovarian cancer the deadliest gynecologic malignancy.^3^ Factors that affect ovarian cancer survival include age and stage at diagnosis, adherence to recommended treatment guidelines, race, socioeconomic status, and rural residence.^4–7^

Rurality may be a contributing barrier to receipt of high-quality ovarian cancer care.^4,7–9^ Rural patients exhibit later stage at diagnosis and shorter survival than their urban counterparts.^10,11^ When compared to urban patients, rural patients have less access to high-volume health care facilities and specialty providers capable of providing the complex care needed for ovarian cancer, which includes surgery, treatment, and disease surveillance.^10,12,13^ Rural patients are also less likely to receive surgery from a gynecologic oncologist, which leads to poorer outcomes, despite this being the guideline recommendations of the National Comprehensive Cancer Network (NCCN).^7,8,14–18^

According to a 2011 report from the Centers for Disease Control and Prevention, over 99% of gynecologic oncologists in the United States work in metropolitan counties, while ∼20% of their patients live in rural counties.^15,19^ Only 13% of gynecologic oncologists work in an area with a population <50,000.^4^ In a survey of gynecologic oncology providers, the majority believed that rural areas have significant barriers to providing optimal cancer care, and that patients should travel to urban cancer centers to receive care within a center of excellence model to receive NCCN guideline therapy from a gynecologic oncology provider.^4,18^

Distance from a patient's residence to a treatment center with gynecologic oncology could represent a significant barrier to care for rural patients and may have a substantial impact on outcomes.^4,20^ In a study performed by Weeks et al., it was shown that rural women traveled 56 miles further than their urban counterparts when receiving ovarian cancer surgical care by a specialist.^14^ The objective of this study was to examine if distance to a gynecologic oncology provider affected survival for patients diagnosed with ovarian cancer in a largely rural midwestern state. Our hypothesis was that those living further from gynecologic oncologists would have worsened cancer survival.

## Methods

### Study population

Data for patients diagnosed with primary malignant (stage I–IV) ovarian cancer (International Classification of Diseases for Oncology, Third Edition [ICD-O-3] C56.9) from 2010 to 2018 were extracted from the Iowa Cancer Registry.^21^ The Iowa Cancer Registry is a population-based cancer registry, which collects information on all cancers diagnosed among Iowa residents, and has been a member of the National Cancer Institute's Surveillance, Epidemiology, and End Results (SEER) Program since 1973.

### Patient-level variables

Tumor-related variables included were American Joint Committee on Cancer 7th edition pathologic Tumor, Node, Metastasis Staging System stage I–IV, grade, ICD-O-3 histology, examination of regional nodes, positive regional nodes, distant metastases at diagnosis, and diagnostic confirmation. Patient-level characteristics included age, race, ethnicity, county of residence at diagnosis, primary insurance payer at diagnosis, marital status at diagnosis, primary surgery site, first course chemotherapy and radiation, vital status, and survival months. Age was categorized as: 20–49, 50–59, 60–69, 70–79, and 80+ years.

Our primary exposure variable, distance to the nearest high-volume health care center with gynecologic oncology, was calculated based on the distance from the centroid of the county of residence at time of diagnosis to centroid of the county, in which the nearest of seven health care centers where gynecologic oncologists practice. These health care centers were chosen due to geographic location either in Iowa or closest proximity to the county in the surrounding states. Based on Registry data, these centers represent the majority of gynecologic oncologists treating the Iowa ovarian cancer cases. Locations of gynecologic oncologists included Des Moines and Iowa City, Iowa; two centers located in Omaha, Nebraska; Rochester, Minnesota; and two centers in Sioux Falls, South Dakota. Distance from the nearest gynecologic oncologist was categorized as: 0–29, 30–49, 50–69, and 70+ miles.

### Community-level variables

Community-level data were extracted from the Health Resources and Services Administration's 2018–2019 Area Health Resource File (AHRF).^22^ The AHRF is a file containing county-level data on demographics, clinicians, and health care facilities. For our analysis, we included the percent of persons below the poverty level, obstetricians/gynecologists, and surgeons per 100,000 population. We calculated percentage of African American non-Hispanic, as well as percentage of Hispanic/Latino residents, from population count data in this file.

Rurality was estimated using 2013 Rural-Urban Continuum Codes (RUCC) for the patient's county of residence at the time of diagnosis.^23^ RUCC codes contain county-level populations and are assigned a code of 1–9, indicating their county's population size and proximity to other densely populated counties. Patients with RUCC codes of 1–3 were categorized as urban, and patients with RUCC codes of 4–9 were categorized as rural.

### Statistical analysis

Demographic, tumor, and treatment characteristics for patients were stratified to assess differences between rural and urban patients. To assess differences in demographic and treatment characteristics, *t*-tests were conducted for continuous variables such as distance to nearest gynecologic oncologist and age. For categorical variables, chi-square analyses were conducted.

To assess the crude association between distance to the nearest gynecologic oncologist and time to death or censorsing, we created Kaplan–Meier curves and conducted Log-Rank tests to compare between groups. Collinearity among variables of interest was determined by collinearity indices >30, and two or more variables with variance decomposition proportions >0.5. Effect modification was assessed by evaluating interactions between our exposure variable and all potential covariates using a 5% significance level. Covariate selection continued after no effect modification was observed and included age, marital status, insurance coverage, stage, grade, radiation, chemotherapy, surgical approach, patient race, patient ethnicity, community percent of residents living in poverty, community percent of African American residents, community percent of Hispanic/Latinx residents, and number of hospital beds and surgeons per 100,000 population.

Dummy variables were used for nominal variables: marital status (married, single, divorced/separated/widowed), insurance coverage (private, Medicaid, Medicare/TRICARE/Veterans Affairs, uninsured), chemotherapy (multiple agent, single agent, recommended but not administered, none), and surgical procedure. Rurality and distance were highly correlated; therefore, we only included distance to specialized care in our models. Covariates were retained in the model if their removal impacted the odds ratio by >10%.

Multivariable Cox proportional hazards models were used to assess changes in survival by time period, adjusted for the aforementioned patient characteristics. The proportional hazards assumption was evaluated by including an interaction variable in our model for each covariate and the log of survival time, and using goodness-of-fit tests to observe correlation between calculated Schonfeld residuals and survival time. In accordance with prevailing standards, survival analyses were restricted to first primary cancers, cases with known age, and those histologically confirmed and followed over time; cases that were identified solely on the basis of death certificates or autopsy reports were excluded.^24,25^ Patients still alive on December 31, 2018, or who had died of other causes were censored. All statistical analyses were completed using SAS 9.4 software (SAS Institute, Cary, NC). This study was exempted from the review of Institutional Review Board.

## Results

There were 1,904 patients who met study inclusion criteria; demographic, tumor, community-level, and treatment characteristics by rurality are given in Table 1. There were 874 (46%) patients who lived in rural areas and 1,030 (54%) who lived in urban areas. Rural patients had a mean (standard deviation [SD]) age of 73.9 (16.0) and urban patients 71.4 (15.8) years (*p* = 0.22). A higher proportion of rural patients were of white race (99% vs. 96%, *p* < 0.001) and non-Hispanic ethnicity (98% vs. 99% *p* = 0.02). A higher proportion of urban patients had private insurance, lived in counties with larger hospitals, and had more surgeons per 100,000 population in their counties than rural patients. A smaller proportion of rural patients lived in areas with more than 11% of the population below poverty level (48% vs. 68%, *p* < 0.001).

**Table 1.** Characteristics of Ovarian Cancer Patients, Stratified by Rural/Urban Status, Iowa Cancer Registry, 2010–2018 (*n* = 1904 Unless Otherwise Specified)

	Rural patients (***n*** = 874), %	Urban patients (***n*** = 1030), %	*p*
Patient-level demographics			
Age at diagnosis (years old)			0.01
20–49	13.1	1	
50–59	17.5	21.7	
60–69	26.3	26.9	
70–79	20.0	19.2	
80+	23.0	17.3	
White race	99.2	96.0	<0.001
Hispanic ethnicity	1.0	2.2	0.02
Insurance coverage			0.004
Medicare/TRICARE/VA	55.9	47.8	
Medicaid	6.7	8.0	
Private insurance	31.0	38.0	
Uninsured	5.6	6.3	
Marital status			0.29
Unmarried	12.0	14.2	
Married	56.5	53.6	
Divorced, separated, widowed	31.6	32.1	
Distance to nearest gynecologic oncologist (gyn/onc), miles (mean, SD)	60.8 (19.6)	33.1 (24.8)	<0.001
0–29 miles from nearest gyn/onc	6.5	58.9	
30–49 miles from nearest gyn/onc	21.0	18.8	
50–69 miles from nearest gyn/onc	38.9	12.8	
70+ miles from nearest gyn/onc	33.7	9.7	
Vital status (% dead)	65.2	57.8	0.001
Community-level characteristics			
Surgeons (per 100,000 population)	5.3 (5.8)	10.2 (11.0)	<0.001
OB/GYNs (per 100,000 population)	2.6 (4.1)	8.8 (9.2)	<0.001
Living in an area with greater than/equal to the average state poverty %^a^	47.7	68.0	<0.001
Living in an area with greater than/equal to the average state % of African American population^b^	6.1	59.6	<0.001
Living in an area with greater than/equal to the average state % of Hispanic population^c^	21.1	46.8	<0.001
Tumor characteristics			
Stage (AJCC 7th edition)			0.33
IA–IC and INOS^d^	24.4	25.9	
IIA–IIC and IINOS^e^	7.2	7.2	
IIIA–IIIC and IIINOS^f^	41.6	44.9	
IV, IVA^g^	25.4	22.4	
Grade			0.84
Well differentiated (I)	12.1	13.6	
Moderately differentiated (II)	15.1	13.8	
Poorly differentiated (III)	30.2	31.1	
Undifferentiated; anaplastic	42.6	41.5	
Treatment			
Surgery			0.03
Resection, unilateral oophorectomy	2.6	2.7	
Bilateral oophorectomy	7.4	7.3	
Oophorectomy with omentectomy	22.2	24.8	
Debulking, cytoreductive surgery, pelvic exenteration	35.6	39.6	
No surgery	32.1	25.3	
Chemotherapy			<0.001
Single agent	2.8	3.0	
Multiple agent	61.6	71.4	
Recommended but not administered	4.7	4.0	
No chemotherapy	30.7	21.8	
Any radiation	1.1	0.8	0.42

^a^ Average state poverty of Iowa is 11%.

^b^ Average percentage of African American population in Iowa is 4%.

^c^ Average percentage of Hispanic population in Iowa is 6%.

^d^ IA–IC and INOS: Stage 1 cancers, including 1A, 1B, and 1C, and stage 1 not otherwise specified.

^e^ IIA–IIC and IINOS: Stage 2 cancers, including 2A, 2B, and 2C, and stage 2 not otherwise specified.

^f^ IIIA–IIIC and IIINOS: Stage 3 cancers, including 3A, 3B, and 3C, and stage 3 not otherwise specified.

^g^ IV, IVA: Stage 4 cancers, including stage 4A.

AJCC, American Joint Committee on Cancer; NOS, not otherwise specified; OB/GYNs, Obstetricians/Gynecologists; SD, standard deviation; VA, Veterans Affairs.

Despite having similar stage and grade distributions, rural patients were less likely to have surgery (25.3% vs. 32.1%, *p* = 0.03) and less likely to receive chemotherapy (21.8% vs. 30.7%, *p* < 0.001) compared to their urban counterparts. Table 2 demonstrates treatment data according to stage at diagnosis. Individuals with later stage cancers were more likely to receive chemotherapy; whereas individuals with earlier stage cancers were more likely to receive surgery.

**Table 2.** Chemotherapy and Surgery Treatment Among Rural Iowa Ovarian Cancer Patients Stratified by Stage, Iowa Cancer Registry, 2010–2018 (*n* = 1562)

	Stage IA–IC and INOS (***N*** = 397)	Stage IIA–IIC and IINOS (***N*** = 122)	Stage IIIA–IIIC and IIINOS (***N*** = 680)	Stage IV and IVA (***N*** = 379)	***p***
	***n* (%)**	***n* (%)**	***n* (%)**	***n* (%)**	
Chemotherapy					<0.001
Single agent	7 (2.0)	1 (1.0)	23 (3.0)	16 (4.0)	
Multiple agent	216 (55.2)	104 (86.7)	531 (78.4)	252 (67.0)	
Recommended but not administered	11 (3.0)	2 (2.0)	27 (4.0)	17 (5.0)	
No chemotherapy	157 (40.1)	13 (10.8)	96 (14.2)	91 (24.2)	
Surgery					<0.001
Resection, unilateral oophorectomy	43 (10.8)	1 (1.0)	2 (<1)	3 (1.0)	
Bilateral oophorectomy	85 (21.4)	16 (13.1)	13 (2.0)	9 (2.0)	
Oophorectomy with omentectomy	215 (54.2)	57 (46.7)	99 (14.6)	31 (8.2)	
Debulking, cytoreductive surgery, pelvic exenteration	48 (12.1)	43 (35.3)	420 (61.2)	139 (36.7)	
No surgery	6 (1.5)	5 (4.1)	146 (21.5)	197 (52.0)	

Of the 1,904 patients with ovarian cancer between 2010 and 2018, 1,562 patients had information on distance to gynecologic oncology (and other relevant covariates) and were included in the subsequent models. The mean (SD) distance to gynecologic oncology was 60.8 (19.6) for rural patients and 33.1 (24.7) for urban patients. Table 3 shows the univariate models and unadjusted associations between travel distance to gynecologic oncology and ovarian cancer survival. These models showed that those who lived further from gynecologic oncology had progressively significantly greater risk of death compared to those who lived 0–29 miles from a gynecologic oncologist.

**Table 3.** Univariate Analysis Assessing Distance to Nearest Gynecologic Oncologist and Survival Among Ovarian Cancer Patients, Iowa Cancer Registry, 2010–2018 (*n* = 1562 Unless Otherwise Specified)

	HR	95% CI
0–29 miles away	(Ref.)	—
30–49 miles away	1.09	1.04–1.15
50–69 miles away	1.19	1.07–1.32
70+ miles away	1.30	1.11–1.51

CI, confidence interval; HR, hazard ratio.

Table 4 shows results from multivariable-adjusted models. In models controlling for age, marital status, stage, county-level poverty, and rate of surgeons per 100,000 population, distance was no longer associated with higher risk of death. More advanced stage, older patient age, and unmarried status were the strongest independent predictors of death during the period of follow-up. Multivariable analysis was also performed without treatment data as it was felt that this could potentially represent another measure of distance and confuse interpretation of results. As there were no meaningful differences in our findings with or without treatment data, treatment data were ultimately included. Other significant factors associated with worsened survival included county-level poverty, with a 8% increased risk of death (hazard ratio [HR] = 1.08, confidence interval [CI]: 0.93–1.24) in counties with 11% or more of the population living below the federal poverty level.

**Table 4.** Multivariable-Adjusted Analysis Assessing Associations of Distance to Nearest Gynecologic Oncologist with Survival Among Ovarian Cancer Patients, Iowa Cancer Registry, 2010–2018 (*n* = 1562 Unless Otherwise Specified)

HRs for variable, controlling for all other variables in the model	HR	95% CI
Vital status (0 = alive, 1 = dead)	—	—
Distance to nearest gynecologic oncologist (exposure), miles away		
0–29	Ref.	—
30–49	1.02	0.96–1.08
50–69	1.04	0.92–1.18
70	1.06	0.89–1.27
Age at diagnosis (years old)		
20–49	Ref.	—
50–59	1.07	0.81–1.40
60–69	1.36	1.05–1.76
70–79	1.59	1.22–2.07
80	2.20	1.64–2.94
Stage		
Stage 1	Ref.	—
Stage 2	4.57	3.09–6.76
Stage 3	9.03	6.47–12.61
Stage 4	12.00	8.47–17.01
Chemotherapy		
No chemotherapy	Ref.	—
Any chemotherapy	0.39	0.33–0.46
Surgery		
Resection/oophorectomy w/or w/o hysterectomy (compared to no surgery)	0.38	0.27–0.55
Oophorectomy w/omentectomy	0.32	0.25–0.41
Cytoreductive surgery/debulking	0.41	0.34–0.49
Community poverty measure		
0–10% persons below poverty line in their community	Ref.	—
11% or greater persons below poverty level in their community	1.08	0.93–1.24
Surgeons per 100,000 population (continuous variable—HR represents increased hazard in 1-unit increase of surgeons)	0.99	0.98–1.00

## Discussion and Conclusions

In this sample of ovarian cancer patients living in a mostly rural state, distance from gynecologic oncology specialists was not associated with risk of death after controlling for demographic and clinical covariates. In unadjusted models, patients with ovarian cancer who lived the farthest (70 miles+) from gynecologic oncology care experienced a 30% increased risk of death, but this was not statistically significant in multivariable adjusted analysis. In adjusted models, later stage at diagnosis and greater age were the strongest predictors of death among our patient cohort. Rurality is known to contribute to worsened prognosis due to later stage at diagnosis.^10^

A large National Cancer Database study also demonstrated that patients with ovarian cancer who live in rural settings with small population and greater distance to tertiary care centers have poorer survival^26^; however, the specific barriers that rural patients encounter after diagnosis that lead to worsened survival have not been fully examined. Results of this study suggest that distance to gynecologic oncology specialists does not make an independent contribution to the worsened survival that rural ovarian cancer patients experience.

Our results support existing evidence that suggests beyond rurality or distance to specialized care, additional complex factors portend a worsened prognosis for ovarian cancer patients. In our study, marital status, stage at diagnosis, county-level poverty, and availability of surgeons were independently associated with ovarian cancer survival. Our analysis also demonstrated that rural patients were less likely to recieve surgery and/or chemotherapy despite similar stage and grade distributions as their urban counterparts.

This finding is consistent with prior studies, which show that administration of adjuvant chemotherapy is 48% lower among rural women as well as being 63% less likely to receive surgery by a gynecologic oncologist.^15,27^ The multiplicity of variables that have been shown to contribute to poorer survival of rural patients suggests that this patient demographic is highly complex. Future work will be needed to further identify contributory variables that may be actionable and help to improve outcomes for rural patients.

Receiving surgery from a specialty trained gynecologic oncologist improves survival for cancer patients.^7–9,16,17,28–30^ Despite this, studies have found that rural patients are less likely to receive surgery by a gynecologic oncologist in a Center of Excellence and are less likely to receive a referral to a specialist by their local diagnosing provider.^7–9,15^ The reasons for this is unclear; it has been suggested that rural general surgeons and Obstetricians/Gynecologist's are more willing and comfortable to perform these surgeries in rural areas.^15^ A study in Utah indicated that gynecologic oncologists see less than half of ovarian cancer patients, with those in rural areas significantly less likely to be seen by gynecologic oncology during the course of their treatment.^7^

Another study demonstrated that those who had surgery performed in a hospital without gynecologic oncology had a higher risk of death than those treated in hospitals with gynecologic oncology services.^9^ Rural patients may also be less likely to actively participate in treatment decisions and are more likely to make decisions based on their local providers' recommendations.^14^ This can lead to decreased understanding of the importance of gynecologic oncology care when the referral to specialist care is not discussed.^14,31,32^ There may also be problems with referral systems, including long wait times for both the referring provider and patient, leading to reduced willingness to engage in the referral process.^8,14,19^

Understanding why rural patients are less likely to receive specialty care for their ovarian cancer treatment is critical to ensuring these patients receive the highest quality care that will afford them the best chance of cancer survival. Unfortunately, our data did not include information about the specialty of the provider that performed surgery or administered chemotherapy, so our results cannot speak directly to this issue.

This study has several strengths and limitations that warrant consideration in interpretation of results. The strengths include that our analyses were based on a population-based, high-quality cancer registry. As a participant in the National Cancer Institute SEER program since 1973, the Iowa Cancer Registry follows the highest standards of quality in the data collection process; this may reduce the potential for misclassification of exposure and outcomes. Finally, as Iowa's population is ∼40% rural,^33^ the large proportion of rural patients allowed us to more easily examine the effect of rurality on cancer survival.^34^ Limitations of this study include that our study population is largely elderly and white, which limits generalizability to more diverse patient populations.^35^ A limitation of our treatment data is that chemotherapy data can be incomplete in cancer registries.

Due to the use of registry data, we were unable to accurately assess individual-level socioeconomic characteristics such as income and education, as these data were not available in the registry. As our study was specifically analyzing patients' county of residence and distance to gynecologic oncology care, a limitation of this study is that we did not assess which patients actually received care by a gynecologic oncologist. Previous studies which also used Iowa Cancer Registry data, conducted enhanced medical record review, which enabled analysis of provider type and receipt of treatment from a gynecologic oncologist.^14,15,27^ Unfortunately, in this study, we were not able to conduct additional medical record review to assess either provider referrals to gynecologic oncologists and/or centers of excellence, the exact location of where they received their care, or whether the patients received treatment that meets standard of care.

This study was designed to assess patients' county of residence and distance to gynecologic oncology care and its impact on survival only. We anticipate that provider referrals will be an important contributor to whether a patient is treated by a gynecologic oncologist at centers of excellence, and that receipt of standard of care from such a provider will be strongly associated with ovarian cancer outcomes.^11,12,14^

Distance to gynecologic care is often thought to be a large barrier to rural ovarian cancer treatment^4,20^; however, we found that it was not a significant determinant of survival for rural patients once other individual and community factors were accounted for. We postulate that referrals and recommendations may impact the rural patients' likelihood to seek more specialized care even if the distance to travel is further.^14,15^ A recent study demonstrated that a gynecologic oncology practice started in a small city with surrounding rural communities significantly reduced travel distance from 48 to 27 miles, improved the percent of people receiving surgery from a gynecologic oncologist from 53% to 97%, and increased median survival time from 31 to 53 months.^36^

This suggests that improving geographic access to gynecologic oncologists can improve survival among rural ovarian cancer patients. Unfortunately, travel to more rural areas in outreach clinics has been viewed as inefficient and burdensome on gynecologic oncologists,^37,38^ and provides insufficient coverage of rural cancer patients.^39^ For these reasons, it has been postulated that increased efforts into telemedicine for gynecologic cancer could decrease or eliminate geographic barriers to high-quality gynecologic cancer care.^37^ In light of the COVID-19 pandemic and increased efforts for telehealth, examination into increased patient access during this time period could be beneficial. Further research is needed to examine barriers to specialized care among rural ovarian cancer patients and elucidate the complex interplay of factors that are causing poorer outcomes.

## Abbreviations Used

AHRF: Area Health Resource File
AJCC: American Joint Committee on Cancer
CI: confidence interval
HR: hazard ratio
NCCN: National Comprehensive Cancer Network
RUCC: Rural-Urban Continuum Codes
SD: standard deviation
SEER: Surveillance, Epidemiology, and End Results
VA: Veterans Affairs
